# Predicting Local Failure after Partial Prostate Re-Irradiation Using a Dosiomic-Based Machine Learning Model

**DOI:** 10.3390/jpm12091491

**Published:** 2022-09-13

**Authors:** Giovanni Pirrone, Fabio Matrone, Paola Chiovati, Stefania Manente, Annalisa Drigo, Alessandra Donofrio, Cristina Cappelletto, Eugenio Borsatti, Andrea Dassie, Roberto Bortolus, Michele Avanzo

**Affiliations:** 1 Medical Physics Department, Centro di Riferimento Oncologico di Aviano (CRO) IRCCS, 33081 Aviano, Italy; 2 Radiation Oncology Department, Centro di Riferimento Oncologico di Aviano (CRO) IRCCS, 33081 Aviano, Italy; 3 Nuclear Medicine Department, Centro di Riferimento Oncologico di Aviano (CRO) IRCCS, 33081 Aviano, Italy

**Keywords:** radiomics, dosiomics, machine learning, radiotherapy, prostate cancer, partial prostate re-irradiation

## Abstract

The aim of this study is to predict local failure after partial prostate re-irradiation for the treatment of isolated locally recurrent prostate cancer by using a machine learning classifier based on radiomic features from pre-treatment computed tomography (CT), positron-emission tomography (PET) and biological effective dose distribution (BED) of the radiotherapy plan. The analysis was conducted on a monocentric dataset of 43 patients with evidence of isolated intraprostatic recurrence of prostate cancer after primary external beam radiotherapy. All patients received partial prostate re-irradiation delivered by volumetric modulated arc therapy. The gross tumor volume (GTV) of each patient was manually contoured from planning CT, choline-PET and dose maps. An ensemble machine learning pipeline including unbalanced data correction and feature selection was trained using the radiomic and dosiomic features as input for predicting occurrence of local failure. The model performance was assessed using sensitivity, specificity, accuracy and area under receiver operating characteristic curves of the score function in 10-fold cross validation repeated 100 times. Local failure was observed in 13 patients (30%), with a median time to recurrence of 36.7 months (range = 6.1–102.4 months). A four variables ensemble machine learning model resulted in accuracy of 0.62 and AUC 0.65. According to our results, a dosiomic machine learning classifier can predict local failure after partial prostate re-irradiation.

## 1. Introduction

Prostate cancer (PCa), the fourth most occurring cancer overall and the second most frequent in men [1], is commonly treated with surgery or radiation therapy (RT), including brachytherapy and external beam radiotherapy (EBRT). Advanced EBRT techniques were associated with a relevant improvement in tumor control and reduced severe toxicity rate, in the last decade [2,3]. However, following radiation treatment, about half patients can develop biochemical recurrence (BCR) of disease diagnosed via a prostate-specific antigen (PSA) test [4]. Additionally, a percentage ranging from 30%–60% of patients with BCR experience an evidence of local disease progression on imaging [5,6]. Despite the existence of guidelines panels, supported by comprehensive literature reviews, there is no consensus on the optimal salvage therapy strategy [7,8]. An available treatment option is represented by partial prostate re-irradiation (PPR), a therapy scheme which combines the use of accurate medical images with stereotactic body radiation therapy (SBRT) to deliver ablative doses to a small region including the tumor volume [9,10].

Heterogeneous disease within and among patients demands a need for an individualized approach to PCa relapse. Precision medicine aims to design treatment plans tailored to the specific disease profile of the patient in order to improve outcomes. However, a major challenge for individualized treatment is accurate prediction of a patient’s disease response to specific therapeutics approach [11]. A widely established method to describe the dose-response after EBRT is the tumor control probability (TCP), a formalism developed to compute the probability of local control by accounting for 3D dose distribution and fractionation [12,13]. However, over the past years, there has been a growing interest in advanced computational methods based on artificial intelligence (AI) [14,15]. In this scenario, radiomics is a powerful tool for applying AI in healthcare. It is a quantitative approach which includes systematic procedures to calculate and analyze high-dimensional data from medical images [16]. The main assumption behind the radiomics concept is that details reflective of the underlying tissue biology, not visible to the human eye, are contained in biomedical images [17]. Through the extraction of mathematical descriptors of intensity and textural information, called radiomic features, the pixel/voxel interrelationships are mined by means of AI methods, mainly machine learning (ML) algorithms, to build a radiomics signature that can predict prognosis, or therapeutic response [18,19]. An extension of radiomics is represented by a more recent approach called dosiomics, where features are extracted from three-dimensional (3D) EBRT dose distribution to encode its spatial and intensity characteristics [20].

A radiomics-based computer-aided decision can be crucial to implementing a personalized salvage program for the treatment of isolated intra-prostatic recurrence in PCa patients who underwent EBRT as primary curative course. Several studies offered a non-invasive way to obtain a diagnostic and risk stratification of PCa patients by means of radiomics in different imaging modalities, mostly involving magnetic resonance imaging (MRI) [21,22,23,24,25,26]. Positron emission tomography (PET), computed tomography (CT) and transrectal ultrasound (TRUS) images have been used to obtain radiomics-based predictive models for PCa detection, therapy outcome, risk group discrimination and RT toxicity [27,28,29,30,31,32]. Moreover, integrating texture analysis of 3D dose distribution of RT can improve model prediction performance of toxicity rates in PCa [33]. Dosiomic features calculated from the target volume were also significantly correlated with biochemical failure in patient with localized PCa who underwent intensity modulated radiation therapy (IMRT) [34]. However, taking into account anatomical, functional and dosimetric information used to perform the PPR therapy scheme can be crucial because the response of re-irradiated tumors could be different respect to those undergoing primary EBRT [35]. At our best knowledge, a model for prediction of response to reirradiation of locally recurrent PCa has not been developed.

The purpose of this work was to build an ML model integrating radiomic and dosiomic features derived from CT, PET and dose distribution in a retrospective cohort of patients with isolated local recurrence of PCa treated with a PPR protocol [36] to be used for prediction of local failure at three years from the end of PPR in a clinical decision support system.

## 2. Materials and Methods

**Figure 1:**
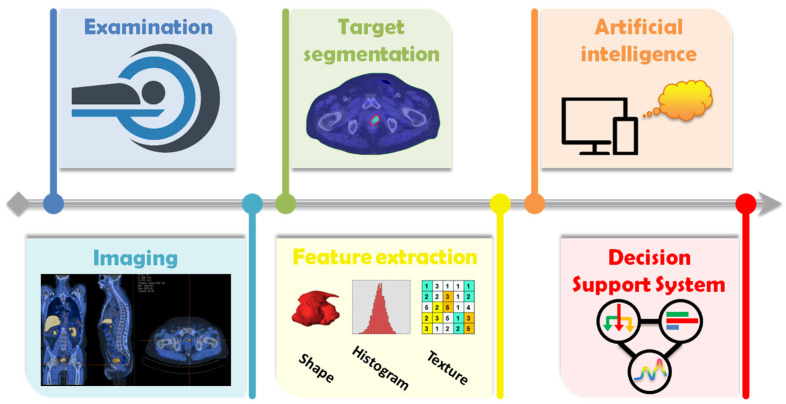
The radiomics framework depicts all the steps needed to build a radiomics model. After medical image acquisition and reconstruction, the region of interest (ROI) is segmented manually, semi-automatically or fully automatically. The feature extraction step, including post-processing of the acquired image, provides numerous quantitative biomarkers managed by means of artificial intelligence to achieve a clinical decision support system.

The radiomics workflow (Figure 1) involves the following steps [37,38]: (i) definition of the clinical endpoint and suitable patient cohort, (ii) identification of appropriate image modalities for feature extraction, (iii) contouring of the region of interest (ROI) segmentation in a manual, semiautomatic or fully automatic manner, (iv) radiomic features extraction from the voxels included in the ROI, (v) training of ML algorithm to obtain a predictive model. The most relevant steps will be highlighted in the following sections.

### 2.1. Patient Data

Forty-three patients with evidence of isolated intra-prostatic recurrence of PCa were treated following a PPR protocol and retrospectively analyzed. All the patients met the following inclusion criteria: (1) previous EBRT as primary treatment, (2) evidence of biochemical recurrence (BCR) defined as a PSA rise of 2 ng/mL above the nadir [39], (3) subsequent detection of isolated local relapse via (11C)-choline PET-CT, and (4) PPR as local salvage treatment. Furthermore, in order to ensure reproducible and repeatable results, we excluded from the analysis all the patients with (11C)-choline PET-CT not acquired in our institution because of inter-scanner variability or different image acquisition protocol.

**Figure 2:**
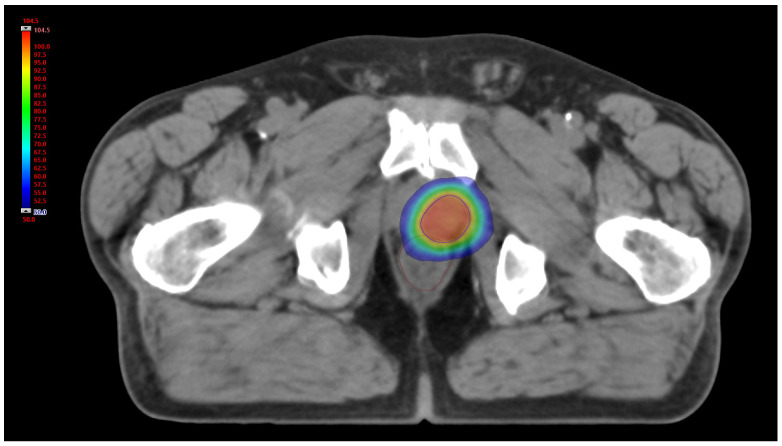
Axial view of 3D relative dose distribution (%). Blue line: PTV; brown line: rectum.

As already extensively described [36], PPR treatment was performed delivering a volumetric modulated arc therapy (VMAT) technique with a course scheme of 35 Gy in seven daily fractions of 5 Gy, five fractions/week (Figure 2); considering an alpha-beta ratio of 1.5 Gy, the total BED at the target was 151.7 Gy [40]. All patients were imaged with a non-contrast-enhanced pre-treatment CT using a 32-slice scanner (Toshiba Aquilion LB, Toshiba Medical Systems Europe, Zoetermeer, the Netherlands). The gross target volume (GTV) was defined with the aid of the (11C)-choline PET as the volume delineated with a semi-automated technique using a fixed threshold of 40% of the maximum signal intensity. To reduce inter-observer variability, minimal manual corrections of the GTV borders were performed to take into account anatomical differences due to possible sub-optimal spatial registration between different image modalities. The dose distributions were calculated on a resolution grid of 2.5 mm or less with Eclipse treatment planning system (TPS) version 13.6, using the Anisotropic Analytical Algorithm version 13.6.23 (Varian Medical System, Palo Alto, CA, USA). For each patient, PET and CT images, together with the dose distributions and GTV contours, were saved in the digital imaging and communications in medicine (DICOM) format. The following analysis, including data conversion, image post processing, feature calculation and model training and validation were performed in Matlab R2021b version: 9.12.0.1927505 R2022a (The MathWorks, Inc., Apple Hill Drive Natick, MA, USA).

Tumor response after PPR was based on PSA level dosage on subsequent follow-up, recorded 3 months after therapy, quarterly for the successive 2 years, biannually until the fifth year and annually thereafter. During follow-up, according to Phoenix Consensum criteria [39], BCR occurred when PSA nadir + 2 ng/mL was observed. In the case of BCR, patients were scanned with an additional (11C)-choline-PET-CT to evaluate for local, regional, or metastatic failure. All recurrences occurred within the treatment field, except for one, which was observed outside the treatment field. Patient characteristics are summarized in Table 1.

### 2.2. Image Processing

It is well known how different choices during features calculation procedures can impact the ML analysis [41,42]. To standardize this practice, we designed an image processing scheme, i.e., the sequence of actions needed to obtain image biomarkers from medical images, following the methodology and definitions of the Image Biomarker Standardization Initiative (IBSI) [43].

Firstly, in order to work with more meaningful information, raw data from PET and dose distribution from TPS were corrected voxel-wise in standard uptake value (SUV) and BED (α/β-ratio of 1.5 Gy), respectively [44]. Because GTV contours within DICOM RTSTRUCT files were stored as a set of points defining a polygon for each image slice, an in-house segmentation algorithm was used to find out which image voxel makes up the binary ROI mask. The chosen method is based on a crossing-number test [45]. It counts the number of times a line starting from the point and going in a fixed direction intersects with the polygonal boundary; all the points with odd counts are considered part of the ROI mask.

PET, CT, BED distribution and the corresponding ROI masks were also interpolated, using a trilinear algorithm, to 1 × 1 × 1 mm^3^ isotropic voxel size to provide a texture features comparable across the cohort and make measurement reproducible [46]. A threshold of 0.5 was used to binarize ROI mask voxels to account for partial volume effects introduced by the trilinear interpolation. Subsequently, since Hounsfield units (HU) do not take non-integer values, CT data were rounded to the nearest integer, whereas no re-segmentation was applied. ROI masks were then utilized to isolate the voxels within the GTV from the surrounding intensities, that were replaced by NaN values.

Voxel intensities within the ROI were discretized into 64 grey levels in order to normalize their values among patients and reduce noise. A fixed bin number discretization approach was preferred because allows a direct comparison of feature values across samples [47]. A total of 380 radiomic features were calculated for each patient using an in-house Matlab code previously benchmarked with different phantoms to quantify the IBSI compliance level [48].

### 2.3. Machine Learning Model

**Figure 3:**
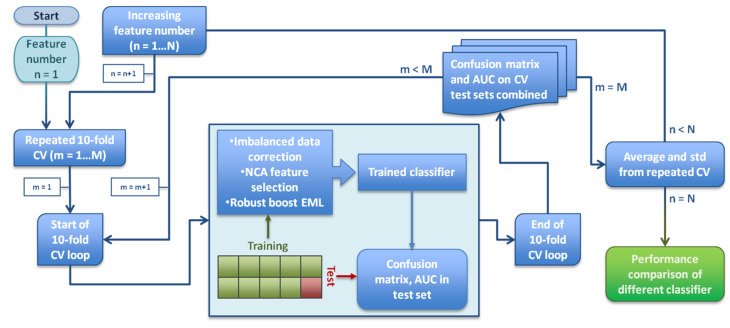
Model building and validation scheme. A repeated 10-fold cross-validation (CV) was performed to yield accuracy and area under curve with standard deviations in order to make model comparisons.

For ML analysis, outcome was binarized considering patient status at three years from the end of the PPR treatment. Local relapse events after PPR were considered as positive whereas relapse-free patients were considered as negative. Radiomic features dataset from PET, CT and BED was used to build an ML model for treatment response classification. As shown in Figure 3, the ML procedure involves three steps: oversampling of the minority class, feature selection and model building from the selected features.

The first step was necessary because local failure rate was 30.2% resulting in an imbalanced dataset. In this condition, the models can be prone to prefer prediction of the dominant class to boost performance during the training phase [49]. To balance the distribution of majority and minority classes, an adjustment strategy using the adaptive synthetic (ADASYN) method was adopted on training data set [50].

Then, feature selection was performed by means of Neighborhood component analysis (NCA) [51], a regularized non-parametric algorithm which learns features’ weights by minimizing an objective function that measures the average leave-one-out classification error. The regularization term, needed to avoid overfitting, was set to 0.02 while the solver type chosen for estimating feature weights was the stochastic gradient descent algorithm.

The final step was outcome modeling by ensemble ML (EML). EML is an approach that seeks better predictive performance by aggregating predictions from multiple weak learners [52]. A well-known system to combine weak learners is boosting, a method which learns them sequentially in an adaptive way: a model at a given step is built giving more importance to observations in the dataset that were previously badly handled. Among ensemble aggregation techniques, RobustBoost was preferred because it is significantly more robust against label noise [53]. A threshold of 5% was used as classification error target, while the maximum number of iterations, often called “learning cycles”, was set to 500. For training the ensemble models, decision trees with a maximum number of split set to 4 were used as weak learners [54].

To yield robust generalized performance of the trained models, a 100-times-repeated 10-fold cross-validation (CV) was performed. During each of the CV iterative loops, the training set (9/10 of the whole dataset) was employed to perform the unbalanced data correction. In this phase, to avoid data leakage [55], the NCA feature selection to obtain the highest weighted variables used to train the EML models was also implemented. Lastly, the testing set (1/10 of the whole dataset) was utilized to obtain predictions by means of the trained classifiers (Figure 3). Furthermore, model performance was evaluated relatively to the increasing number of features. With this goal, the CVs were run increasing the variable number up to a maximum of 8 sorted in the feature selection process.

The model was assessed by estimating sensitivity (true positive rate), specificity (true negative rate), accuracy (fraction of the events correctly classified) and the area under curve (AUC) from the receiver operating characteristic (ROC) analysis [56]. To obtain a global model performance, average values and standard deviations (σ) of these scores were computed over all the repeated CVs. At the end, the best predictive classifier was considered as the one with the highest accuracy.

## 3. Results

**Figure 4:**
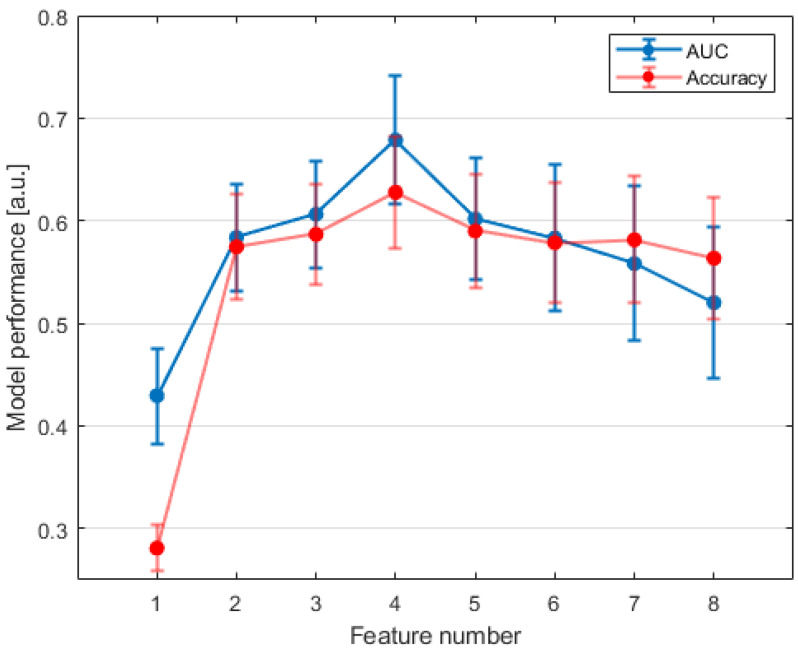
Model performance as a function of the increasing number of features.

**Figure 5:**
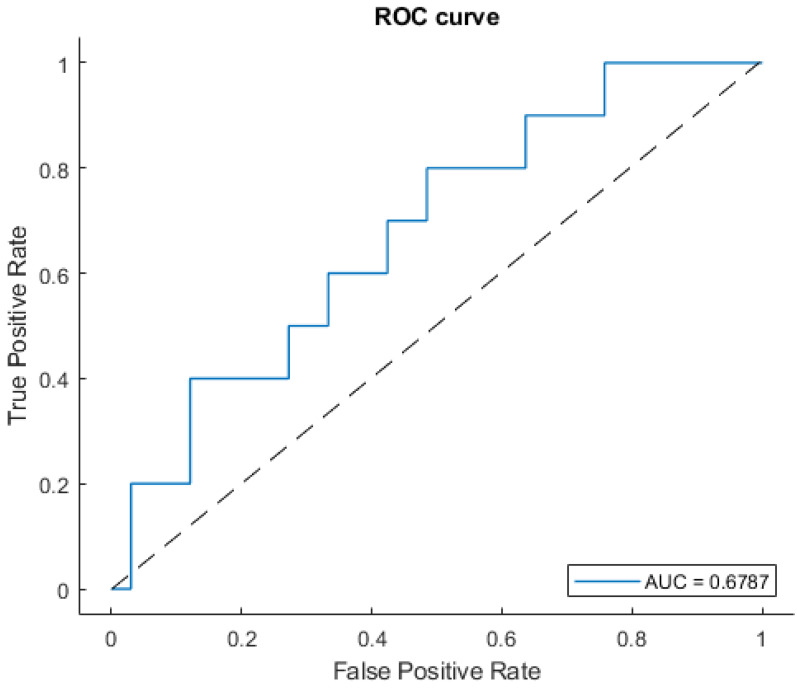
ROC curve of the selected EML model after the 100-times-repeated 10-fold CV.

**Figure 6:**
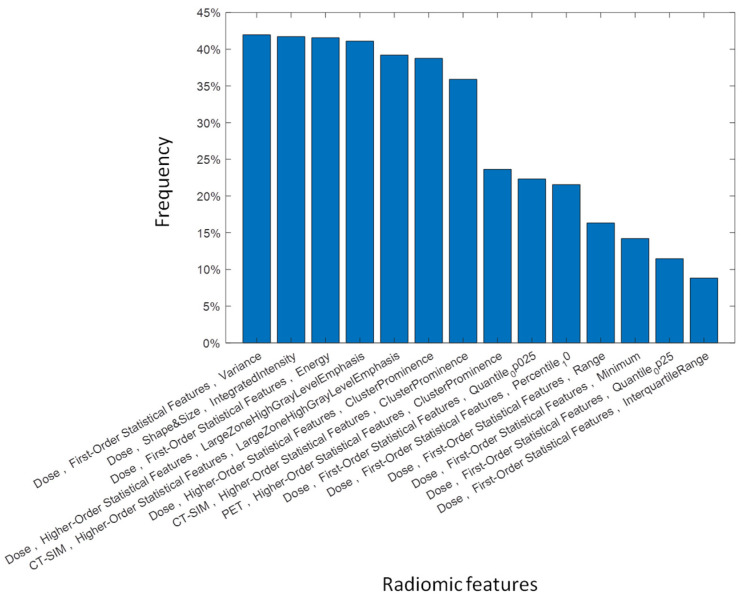
Histogram of the most frequently selected variables in the neighborhood component analysis feature selection during the 100-times-repeated 10-fold CV.

Model scores obtained which increased the feature number are shown in Figure 4. The model with best performance was built by a radiomic signature based on four variables, showing an AUC of 0.68 (±0.06 σ), with a sensitivity, specificity, and accuracy of 0.61 (±0.11 σ), 0.63 (±0.06 σ), and 0.63 (±0.06 σ) respectively. The ROC curve of the selected EML and the most frequently chosen radiomic features during repeated CV are shown in Figure 5 and Figure 6, respectively. Two predictors, “Variance” and “Energy”, were intensity-based statistical features, while “Integrated Intensity” and “Large zone high gray level emphasis” were morphological and textural features, respectively [43]. All the variables were extracted from BED distributions.

## 4. Discussion

Radiomics has recently emerged as a tool for image analysis which converts medical images into quantitative descriptors [57]. PCa radiomics has been largely applied, mostly using multiparametric MRI, for automated and non-invasive tumor localization and characterization [58]. Treatment response prediction by radiomic features was also the aim of several studies. For instance, Alongi et al. [28] demonstrated how radiomic features from PET/CT imaging are associated with PCa patients’ outcome. Similarly, Abdollahi at al. developed a model based on MRI radiomic features to predict EBRT response, Gleason Score and PCa stage [59]. Since the use of imaging is vital to diagnose and stage recurrence, in particular using multiparametric MRI and PET/CT [60], it can be postulated that radiomics analysis could provide useful information to predict its outcome. In particular, PET/CT is known to provide useful data regarding the metabolic and morphologic phenotype of the recurrent tumor. BED distribution was found to accurately describe the radiotherapy treatment: R. J. Klement et al. correlated the near-minimum and near-maximum dose prescribed to the planning target volume and their average to the risk of local recurrence of non-small cell lung cancer [61].

The inclusion of BED maps delivered during EBRT is an effective way for improving the predictive power of AI-based classifiers. Avanzo et al. [62] highlighted this point by showing how the integration of radiomic and machine-learned predictors from BED maps is advantageous, relative to the use of only CT-derived features, in increasing the predictive performances of both ML and deep learning models. Likewise, Welch et al. [63] demonstrated the effectiveness of the aggregation of features derived from different kinds of data (in this case clinical, radiomic and dosiomics) to predict the loco-regional failure of head and neck cancer.

As such, the purpose of this study was to explore the potential of imaging and dose data of recurrent PCa to for predicting treatment response to EBRT, where the endpoint for response was a new clinical recurrence at three years from the end of the therapy. The obtained results, although the model performance on the repeated validation was only fair, demonstrates that radiomics-based ML models can help in identifying recurrent tumors at highest risk of lack of response.

As is well known, overfitting is a typical fault of ML models, meaning that the model precisely follows the data random noise instead of detecting patterns or trends of data [64]. To build the radiomic signature and prevent overfitting, the most meaningful variables were chosen by using NCA feature selection on CV (Figure 3). The radiomic model with best performance showed an accuracy of 0.62 (±0.06 σ) and was derived by four radiomic features which were all selected from the dose distribution. Because of this result, the classifier can be considered as a dosiomic model, suggesting that local failure-free survival is strictly correlated to the BED pattern within the RT target. The four most frequently selected dosiomic variables were: “Variance” and “Energy”, two first order statistical features describing the spread and magnitude of BED distribution in the tumor; “Integrated intensity”, the average dose intensity in the GTV multiplied by its volume; “Large Zone High Gray Level Emphasis” (LZHGE) that describes the emphasis of high-level BED zones. These results are in agreement with previous findings where the highest BED was proven to increase relapse-free survival after brachytherapy [65]. Dose escalation has been shown to improve the response of PCa. Moreover, PCa is believed to be sensitive to fractionation schedule, also suggesting the superiority of BED over dose for treatment analysis [66].

Our study has several limitations. Although inclusion criteria produced a homogeneous patient cohort, this resulted in a small dataset. In order to overcome this issue, a CV approach was implemented to assess a more robust model performance. Moreover, the result could be affected by selection bias because of the retrospective nature of the study. Another restriction of this work is the relative short follow-up of the patient cohort; this implies that patients with late relapse are categorized wrongly as relapse-free. Therefore, short follow-up can represent a confounding factor for predictive analyses. Finally, the patient population was imbalanced in favor of relapse-free patients. Despite mitigating the effect of imbalance by using ADASYN algorithm, the model would benefit from a more balanced dataset. A promising strategy to further increase the predictive power is to widely investigate the patient dataset. Other clinical factors or features, such as those extracted from genomes can also be found to be associated with isolated local recurrence incidence after PPR, providing a deeper knowledge [67]. Therefore, their integration into the model can improve the prediction capability and robustness.

Despite its limitations, to the best of our knowledge, this study offers a proof-of-concept of using an ML radiomics-based model to predict treatment response after reirradiation of a radio-recurrent PCa. A particularly attractive process in which our ML approach could find its application is the tumor dose painting (DP) [68,69]. It is well known that the common planning process involves patient preparation, simulation CT-scan, target and organ at risk delineation and dose distribution calculation; all these steps are used to create an individualized radiotherapy plan according to the treatment prescription. Instead of prescribing a standard dose, it could be possible to apply a DP procedure where the prescribed dose is adapted according to the presumed BED spatial dependences within the tumor found by our predictive model. These findings open new scenarios for developing decision support systems able to help experts in defining the better salvage strategy for managing recurrent tumors.

## 5. Conclusions

A dosiomic-based classifier was developed to predict therapy response at three years from the end of PPR. Dose-based predictors contain important details related to patient outcome. Future developments such as an inter-scanner calibration and an external validation are needed prior its inclusion into clinical practice.

## Figures and Tables

**Table 1:** Characteristics of patient cohort.

Characteristics	Value
Patient number	43
Age at PPR (years), median (range)	77.5 (57–90)
Follow-up (months), median (range)	36.7 (6.1–102.4)
PSA before PPR (ng/mL), median (range)	3.2 (0.09–16.5)
PSA after PPR (ng/mL), median (range)	1.1 (0.07–12.08)
Interval between PPR and BCR (months), median (range)	30.1 (7.9–51.8)
LF after PPR	13 (30%)
In-field	10 (23%)
Out-field	1 (2%)
In-field/Out-field	2 (5%)
PSA at LF detection after PPR (ng/mL), median (range)	4.8 (1.8–27)

Abbreviations: PSA—prostate-specific antigen; BCR—biochemical recurrence; PPR—partial prostate re-irradiation; LF—local failure.

## Data Availability

Data available on request due to privacy/ethical restrictions.

## References

[1] Sung H., Ferlay J., Siegel R.L., Laversanne M., Soerjomataram I., Jemal A., Bray F. (2021). Global Cancer Statistics 2020: GLOBOCAN Estimates of Incidence and Mortality Worldwide for 36 Cancers in 185 Countries. CA Cancer J. Clin..

[2] Zelefsky M.J., Kollmeier M., Cox B., Fidaleo A., Sperling D., Pei X., Carver B., Coleman J., Lovelock M., Hunt M. (2012). Improved Clinical Outcomes with High-Dose Image Guided Radiotherapy Compared with Non-IGRT for the Treatment of Clinically Localized Prostate Cancer. Int. J. Radiat. Oncol. Biol. Phys..

[3] Kuban D.A., Levy L.B., Cheung M.R., Lee A.K., Choi S., Frank S., Pollack A. (2011). Long-Term Failure Patterns and Survival in a Randomized Dose-Escalation Trial for Prostate Cancer. Who Dies of Disease?. Int. J. Radiat. Oncol..

[4] Goy B.W., Burchette R., Soper M.S., Chang T., Cosmatos H.A. (2020). Ten-Year Treatment Outcomes of Radical Prostatectomy Vs External Beam Radiation Therapy Vs Brachytherapy for 1503 Patients with Intermediate-Risk Prostate Cancer. Urology.

[5] Jansen B.H.E., van Leeuwen P.J., Wondergem M., van der Sluis T.M., Nieuwenhuijzen J.A., Knol R.J.J., van Moorselaar R.J.A., van der Poel H.G., Oprea-Lager D.E., Vis A.N. (2021). Detection of Recurrent Prostate Cancer Using Prostate-Specific Membrane Antigen Positron Emission Tomography in Patients Not Meeting the Phoenix Criteria for Biochemical Recurrence After Curative Radiotherapy. Eur. Urol. Oncol..

[6] Raveenthiran S., Yaxley J., Gianduzzo T., Kua B., McEwan L., Wong D., Tsang G., MacKean J. (2019). The Use of 68Ga-PET/CT PSMA to Determine Patterns of Disease for Biochemically Recurrent Prostate Cancer Following Primary Radiotherapy. Prostate Cancer Prostatic Dis..

[7] Marra G., Valerio M., Emberton M., Heidenreich A., Crook J.M., Bossi A., Pisters L.L. (2019). Salvage Local Treatments After Focal Therapy for Prostate Cancer. Eur. Urol. Oncol..

[8] Van den Broeck T., van den Bergh R.C.N., Briers E., Cornford P., Cumberbatch M., Tilki D., De Santis M., Fanti S., Fossati N., Gillessen S. (2020). Biochemical Recurrence in Prostate Cancer: The European Association of Urology Prostate Cancer Guidelines Panel Recommendations. Eur. Urol. Focus.

[9] Alongi F., De Bari B., Campostrini F., Arcangeli S., Matei D.V., Lopci E., Petralia G., Bellomi M., Chiti A., Magrini S.M. (2013). Salvage Therapy of Intraprostatic Failure after Radical External-Beam Radiotherapy for Prostate Cancer: A Review. Crit. Rev. Oncol. Hematol..

[10] Lo S.S., Fakiris A.J., Chang E.L., Mayr N.A., Wang J.Z., Papiez L., Teh B.S., McGarry R.C., Cardenes H.R., Timmerman R.D. (2010). Stereotactic Body Radiation Therapy: A Novel Treatment Modality. Nat. Rev. Clin. Oncol..

[11] Lambin P., van Stiphout R.G.P.M., Starmans M.H.W., Rios-Velazquez E., Nalbantov G., Aerts H.J.W.L., Roelofs E., van Elmpt W., Boutros P.C., Granone P. (2013). Predicting Outcomes in Radiation Oncology—Multifactorial Decision Support Systems. Nat. Rev. Clin. Oncol..

[12] Sachpazidis I., Mavroidis P., Zamboglou C., Klein C.M., Grosu A.-L., Baltas D. (2020). Prostate Cancer Tumour Control Probability Modelling for External Beam Radiotherapy Based on Multi-Parametric MRI-GTV Definition. Radiat. Oncol..

[13] Royce T.J., Mavroidis P., Wang K., Falchook A.D., Sheets N.C., Fuller D.B., Collins S.P., Naqa I.E., Song D.Y., Ding G.X. (2021). Tumor Control Probability Modeling and Systematic Review of the Literature of Stereotactic Body Radiation Therapy for Prostate Cancer. Int. J. Radiat. Oncol. Biol. Phys..

[14] Hamet P., Tremblay J. (2017). Artificial Intelligence in Medicine. Metabolism.

[15] Parekh V.S., Jacobs M.A. (2019). Deep Learning and Radiomics in Precision Medicine. Expert Rev. Precis. Med. Drug Dev..

[16] Avanzo M., Stancanello J., El Naqa I. (2017). Beyond Imaging: The Promise of Radiomics. Phys. Med..

[17] Mannil M., von Spiczak J., Manka R., Alkadhi H. (2018). Texture Analysis and Machine Learning for Detecting Myocardial Infarction in Noncontrast Low-Dose Computed Tomography: Unveiling the Invisible. Investig. Radiol..

[18] Lambin P., Leijenaar R.T.H., Deist T.M., Peerlings J., de Jong E.E.C., van Timmeren J., Sanduleanu S., Larue R.T.H.M., Even A.J.G., Jochems A. (2017). Radiomics: The Bridge between Medical Imaging and Personalized Medicine. Nat. Rev. Clin. Oncol..

[19] Aerts H.J.W.L., Velazquez E.R., Leijenaar R.T.H., Parmar C., Grossmann P., Carvalho S., Bussink J., Monshouwer R., Haibe-Kains B., Rietveld D. (2014). Decoding Tumour Phenotype by Noninvasive Imaging Using a Quantitative Radiomics Approach. Nat. Commun..

[20] Avanzo M., Pirrone G., Vinante L., Caroli A., Stancanello J., Drigo A., Massarut S., Mileto M., Urbani M., Trovo M. (2020). Electron Density and Biologically Effective Dose (BED) Radiomics-Based Machine Learning Models to Predict Late Radiation-Induced Subcutaneous Fibrosis. Front. Oncol..

[21] Khalvati F., Zhang J., Chung A.G., Shafiee M.J., Wong A., Haider M.A. (2018). MPCaD: A Multi-Scale Radiomics-Driven Framework for Automated Prostate Cancer Localization and Detection. BMC Med. Imaging.

[22] Cameron A., Khalvati F., Haider M.A., Wong A. (2016). MAPS: A Quantitative Radiomics Approach for Prostate Cancer Detection. IEEE Trans. Biomed. Eng..

[23] Sidhu H.S., Benigno S., Ganeshan B., Dikaios N., Johnston E.W., Allen C., Kirkham A., Groves A.M., Ahmed H.U., Emberton M. (2017). Textural Analysis of Multiparametric MRI Detects Transition Zone Prostate Cancer. Eur. Radiol..

[24] Bleker J., Kwee T.C., Dierckx R.A.J.O., de Jong I.J., Huisman H., Yakar D. (2020). Multiparametric MRI and Auto-Fixed Volume of Interest-Based Radiomics Signature for Clinically Significant Peripheral Zone Prostate Cancer. Eur. Radiol..

[25] Woźnicki P., Westhoff N., Huber T., Riffel P., Froelich M.F., Gresser E., von Hardenberg J., Mühlberg A., Michel M.S., Schoenberg S.O. (2020). Multiparametric MRI for Prostate Cancer Characterization: Combined Use of Radiomics Model with PI-RADS and Clinical Parameters. Cancers.

[26] Wang J., Wu C.-J., Bao M.-L., Zhang J., Wang X.-N., Zhang Y.-D. (2017). Machine Learning-Based Analysis of MR Radiomics Can Help to Improve the Diagnostic Performance of PI-RADS v2 in Clinically Relevant Prostate Cancer. Eur. Radiol..

[27] Zamboglou C., Bettermann A.S., Gratzke C., Mix M., Ruf J., Kiefer S., Jilg C.A., Benndorf M., Spohn S., Fassbender T.F. (2021). Uncovering the Invisible-Prevalence, Characteristics, and Radiomics Feature-Based Detection of Visually Undetectable Intraprostatic Tumor Lesions in 68GaPSMA-11 PET Images of Patients with Primary Prostate Cancer. Eur. J. Nucl. Med. Mol. Imaging.

[28] Alongi P., Laudicella R., Stefano A., Caobelli F., Comelli A., Vento A., Sardina D., Ganduscio G., Toia P., Ceci F. (2020). Choline PET/CT Features to Predict Survival Outcome in High Risk Prostate Cancer Restaging: A Preliminary Machine-Learning Radiomics Study. Q. J. Nucl. Med. Mol. Imaging.

[29] Osman S.O.S., Leijenaar R.T.H., Cole A.J., Lyons C.A., Hounsell A.R., Prise K.M., O’Sullivan J.M., Lambin P., McGarry C.K., Jain S. (2019). Computed Tomography-Based Radiomics for Risk Stratification in Prostate Cancer. Int. J. Radiat. Oncol. Biol. Phys..

[30] Mostafaei S., Abdollahi H., Kazempour Dehkordi S., Shiri I., Razzaghdoust A., Zoljalali Moghaddam S.H., Saadipoor A., Koosha F., Cheraghi S., Mahdavi S.R. (2020). CT Imaging Markers to Improve Radiation Toxicity Prediction in Prostate Cancer Radiotherapy by Stacking Regression Algorithm. Radiol. Med..

[31] Zhang Q., Xiong J., Cai Y., Shi J., Xu S., Zhang B. (2020). Multimodal Feature Learning and Fusion on B-Mode Ultrasonography and Sonoelastography Using Point-Wise Gated Deep Networks for Prostate Cancer Diagnosis. Biomed. Tech..

[32] Huang X., Chen M., Liu P., Du Y. (2020). Texture Feature-Based Classification on Transrectal Ultrasound Image for Prostatic Cancer Detection. Comput. Math. Methods Med..

[33] Rossi L., Bijman R., Schillemans W., Aluwini S., Cavedon C., Witte M., Incrocci L., Heijmen B. (2018). Texture Analysis of 3D Dose Distributions for Predictive Modelling of Toxicity Rates in Radiotherapy. Radiother. Oncol. J. Eur. Soc. Ther. Radiol. Oncol..

[34] Murakami Y., Soyano T., Kozuka T., Ushijima M., Koizumi Y., Miyauchi H., Kaneko M., Nakano M., Kamima T., Hashimoto T. (2022). Dose-Based Radiomic Analysis (Dosiomics) for Intensity Modulated Radiation Therapy in Patients with Prostate Cancer: Correlation Between Planned Dose Distribution and Biochemical Failure. Int. J. Radiat. Oncol..

[35] Nix M., Gregory S., Aldred M., Aspin L., Lilley J., Al-Qaisieh B., Uzan J., Svensson S., Dickinson P., Appelt A.L. (2022). Dose Summation and Image Registration Strategies for Radiobiologically and Anatomically Corrected Dose Accumulation in Pelvic Re-Irradiation. Acta Oncol..

[36] Matrone F., Revelant A., Fanetti G., Polesel J., Chiovati P., Avanzo M., De Renzi F., Colombo C.B., Arcicasa M., De Paoli A. (2021). Partial Prostate Re-Irradiation for the Treatment of Isolated Local Recurrence of Prostate Cancer in Patients Previously Treated with Primary External Beam Radiotherapy: Short-Term Results of a Monocentric Study. Neoplasma.

[37] Papanikolaou N., Matos C., Koh D.M. (2020). How to Develop a Meaningful Radiomic Signature for Clinical Use in Oncologic Patients. Cancer Imaging.

[38] Shur J.D., Doran S.J., Kumar S., Ap Dafydd D., Downey K., O’Connor J.P.B., Papanikolaou N., Messiou C., Koh D.-M., Orton M.R. (2021). Radiomics in Oncology: A Practical Guide. Radiogr. Rev. Publ. Radiol. Soc. N. Am. Inc..

[39] Roach M., Hanks G., Thames H., Schellhammer P., Shipley W.U., Sokol G.H., Sandler H. (2006). Defining Biochemical Failure Following Radiotherapy with or without Hormonal Therapy in Men with Clinically Localized Prostate Cancer: Recommendations of the RTOG-ASTRO Phoenix Consensus Conference. Int. J. Radiat. Oncol. Biol. Phys..

[40] Jones B., Dale R.G., Deehan C., Hopkins K.I., Morgan D.A. (2001). The Role of Biologically Effective Dose (BED) in Clinical Oncology. Clin. Oncol. R. Coll. Radiol. G. B..

[41] Hatt M., Tixier F., Pierce L., Kinahan P.E., Le Rest C.C., Visvikis D. (2017). Characterization of PET/CT Images Using Texture Analysis: The Past, the Present … Any Future?. Eur. J. Nucl. Med. Mol. Imaging.

[42] Baeßler B., Weiss K., Pinto dos Santos D. (2019). Robustness and Reproducibility of Radiomics in Magnetic Resonance Imaging: A Phantom Study. Investig. Radiol..

[43] Zwanenburg A., Vallières M., Abdalah M.A., Aerts H.J.W.L., Andrearczyk V., Apte A., Ashrafinia S., Bakas S., Beukinga R.J., Boellaard R. (2020). The Image Biomarker Standardization Initiative: Standardized Quantitative Radiomics for High-Throughput Image-Based Phenotyping. Radiology.

[44] Avanzo M., Barbiero S., Trovo M., Bissonnette J.-P., Jena R., Stancanello J., Pirrone G., Matrone F., Minatel E., Cappelletto C. (2017). Voxel-by-Voxel Correlation between Radiologically Radiation Induced Lung Injury and Dose after Image-Guided, Intensity Modulated Radiotherapy for Lung Tumors. Phys. Medica.

[45] Shimrat M. (1962). Algorithm 112: Position of Point Relative to Polygon. Commun. ACM.

[46] Coroller T.P., Agrawal V., Narayan V., Hou Y., Grossmann P., Lee S.W., Mak R.H., Aerts H.J.W.L. (2016). Radiomic Phenotype Features Predict Pathological Response in Non-Small Cell Lung Cancer. Radiother. Oncol. J. Eur. Soc. Ther. Radiol. Oncol..

[47] Yip S.S., Aerts H.J.W.L. (2016). Applications and Limitations of Radiomics. Phys. Med. Biol..

[48] Bettinelli A., Marturano F., Avanzo M., Loi E., Menghi E., Mezzenga E., Pirrone G., Sarnelli A., Strigari L., Strolin S. (2022). A Novel Benchmarking Approach to Assess the Agreement among Radiomic Tools. Radiology.

[49] Lv J., Chen X., Liu X., Du D., Lv W., Lu L., Wu H. (2022). Imbalanced Data Correction Based PET/CT Radiomics Model for Predicting Lymph Node Metastasis in Clinical Stage T1 Lung Adenocarcinoma. Front. Oncol..

[50] He H., Bai Y., Garcia E.A., Li S. ADASYN: Adaptive Synthetic Sampling Approach for Imbalanced Learning. Proceedings of the 2008 IEEE International Joint Conference on Neural Networks (IEEE World Congress on Computational Intelligence).

[51] Yang W., Wang K., Zuo W. (2012). Neighborhood Component Feature Selection for High-Dimensional Data. J. Comput..

[52] Zhang C., Ma Y. (2012). Ensemble Machine Learning, Methods and Applications.

[53] Freund Y. (2009). A More Robust Boosting Algorithm. arXiv.

[54] Loh W.-Y. (2011). Classification and Regression Trees. WIREs Data Min. Knowl. Discov..

[55] Parmar C., Barry J.D., Hosny A., Quackenbush J., Aerts H.J.W.L. (2018). Data Analysis Strategies in Medical Imaging. Clin. Cancer Res. Off. J. Am. Assoc. Cancer Res..

[56] Kumar R., Indrayan A. (2011). Receiver Operating Characteristic (ROC) Curve for Medical Researchers. Indian Pediatr..

[57] Lambin P., Rios-Velazquez E., Leijenaar R., Carvalho S., van Stiphout R.G.P.M., Granton P., Zegers C.M.L., Gillies R., Boellard R., Dekker A. (2012). Radiomics: Extracting More Information from Medical Images Using Advanced Feature Analysis. Eur. J. Cancer.

[58] Midiri F., Vernuccio F., Purpura P., Alongi P., Bartolotta T.V. (2021). Multiparametric MRI and Radiomics in Prostate Cancer: A Review of the Current Literature. Diagnostics.

[59] Abdollahi H., Mofid B., Shiri I., Razzaghdoust A., Saadipoor A., Mahdavi A., Galandooz H.M., Mahdavi S.R. (2019). Machine Learning-Based Radiomic Models to Predict Intensity-Modulated Radiation Therapy Response, Gleason Score and Stage in Prostate Cancer. Radiol. Med..

[60] Shaikh F., Dupont-Roettger D., Dehmeshki J., Kubassova O., Quraishi M.I. (2020). Advanced Imaging of Biochemical Recurrent Prostate Cancer With PET, MRI, and Radiomics. Front. Oncol..

[61] Klement R.J., Sonke J.-J., Allgäuer M., Andratschke N., Appold S., Belderbos J., Belka C., Blanck O., Dieckmann K., Eich H.T. (2020). Correlating Dose Variables with Local Tumor Control in Stereotactic Body Radiation Therapy for Early-Stage Non-Small Cell Lung Cancer: A Modeling Study on 1500 Individual Treatments. Int. J. Radiat. Oncol. Biol. Phys..

[62] Avanzo M., Gagliardi V., Stancanello J., Blanck O., Pirrone G., El Naqa I., Revelant A., Sartor G. (2021). Combining Computed Tomography and Biologically Effective Dose in Radiomics and Deep Learning Improves Prediction of Tumor Response to Robotic Lung Stereotactic Body Radiation Therapy. Med. Phys..

[63] Welch M.L., McIntosh C., McNiven A., Huang S.H., Zhang B.-B., Wee L., Traverso A., O’Sullivan B., Hoebers F., Dekker A. (2020). User-Controlled Pipelines for Feature Integration and Head and Neck Radiation Therapy Outcome Predictions. Phys. Med. Eur. J. Med. Phys..

[64] Parekh V., Jacobs M.A. (2016). Radiomics: A New Application from Established Techniques. Expert Rev. Precis. Med. Drug Dev..

[65] Stock R.G., Stone N.N., Cesaretti J.A., Rosenstein B.S. (2006). Biologically Effective Dose Values for Prostate Brachytherapy: Effects on PSA Failure and Posttreatment Biopsy Results. Int. J. Radiat. Oncol..

[66] Zaorsky N.G., Palmer J.D., Hurwitz M.D., Keith S.W., Dicker A.P., Den R.B. (2015). What Is the Ideal Radiotherapy Dose to Treat Prostate Cancer? A Meta-Analysis of Biologically Equivalent Dose Escalation. Radiother. Oncol..

[67] Castaldo R., Cavaliere C., Soricelli A., Salvatore M., Pecchia L., Franzese M. (2021). Radiomic and Genomic Machine Learning Method Performance for Prostate Cancer Diagnosis: Systematic Literature Review. J. Med. Internet Res..

[68] Ling C.C., Humm J., Larson S., Amols H., Fuks Z., Leibel S., Koutcher J.A. (2000). Towards Multidimensional Radiotherapy (MD-CRT): Biological Imaging and Biological Conformality. Int. J. Radiat. Oncol..

[69] Vaugier L., Ferrer L., Mengue L., Jouglar E. (2020). Radiomics for Radiation Oncologists: Are We Ready to Go?. BJR Open.

